# Activation of Notch3 promotes pulmonary arterial smooth muscle cells proliferation via Hes1/p27Kip1 signaling pathway

**DOI:** 10.1016/j.fob.2015.08.007

**Published:** 2015-08-12

**Authors:** Yang Song, Yonghong Zhang, Haoxiang Jiang, Yanting Zhu, Lu Liu, Wei Feng, Lan Yang, Yibin Wang, Manxiang Li

**Affiliations:** a Department of Respiratory Medicine, The First Affiliated Hospital of Xi’an Jiaotong University, Xi’an, Shaanxi 710061, PR China; b Department of Radiology, Xi’an Children’s Hospital, Xi’an, Shaanxi 710003, PR China; c Departments of Anesthesiology, Physiology, and Medicine, David Geffen School of Medicine, University of California at Los Angeles, Los Angeles, CA 90095, USA

**Keywords:** PAH, pulmonary arterial hypertension, PAP, pulmonary arterial pressure, PASMCs, pulmonary arterial smooth muscle cells, PASMCs, Proliferation, NICD3, Hes1, P27Kip1

## Abstract

Activation of the Notch3 cascade is involved in the development of pulmonary arterial hypertension by stimulating the proliferation of vascular smooth muscle cells. However, the detailed molecular mechanisms underlying this effect are still unclear. The present study aims to address this issue. We demonstrated that over-expression of intracellular domain of the Notch3 receptor (NICD3) by adenovirus transfection dramatically induced proliferation of primary cultured pulmonary artery smooth muscle cells. This was accompanied with up-regulation of Hes1 protein and down-regulation of p27Kip1 protein. More importantly, we observed that prior silencing of Hes1 with siRNA blocked NICD3 over-expression-induced p27Kip1 reduction and cell proliferation. The present study suggests that Hes1 lies downstream of NICD3 and particularly mediates Notch3 signaling-induced proliferation of pulmonary arterial smooth muscle cells by down-regulation of p27Kip1 expression.

## Highlights

• Notch3 receptor (NICD3) over-expression induces proliferation of pulmonary arterial smooth muscle cells (PASMCs).
• NICD3 up-regulates Hes1 expression and reduces p27Kip1 expression.
• Hes1 mediates NICD3-induced p27Kip1 reduction and proliferation of PASMCs.

## 1 Introduction

Pulmonary arterial hypertension (PAH) is a clinical syndrome characterized by sustained elevation of pulmonary arterial resistance and pulmonary arterial pressure (PAP) leading to right heart failure and death [1,2]. Pulmonary arterial remodeling, occurring mostly in the distal pulmonary arteries, is a hallmark of all types of PAH [3]. Excessive proliferation of pulmonary arterial smooth muscle cells (PASMCs) is critical in the pathogenesis of pulmonary artery remodeling [4]. During the transition of PASMCs from quiescent to proliferative types, multiple signaling cascades are activated and mediate the phenotype change [5,6]. Therefore, it is important to clarify these pathways and exploring key targets to suppress PASMCs proliferation.

Notch signaling is a highly conserved cascade and plays an important role in determining cell proliferation and apoptosis [7]. Notch is a group of transmembrane receptors consisting 4 members (Notch 1–4), the ligand of Notch consists of 5 members JAGGED 1/2 and DELTA 1/3/4 [8]. Upon ligand binding, the Notch receptor is activated by two sequential steps of proteolytical cleavage. These processes release the intracellular domain of the Notch receptor (NICD), which translocates into the nucleus and works with the DNA-binding factor, CBF1/RBPJ to regulate the transcription of particular target genes [9]. It has been shown that Hairy-and-enhancer of split (Hes) and Hairy/Enhancer of Split-related (Hrt; also known as Hey) gene families are down-stream targets of NICD [10], which belong to the basic helix-loop-helix (bHLH) transcriptional regulators and repress the transcription of target genes such as p57 kip2 [11], p27Kip1 [12] and p21waf1/cip1 [13] and affect cell fate decision.

Recent studies have shown that activation of Notch3 signaling pathway is involved in the development of PAH by stimulation of PASMCs proliferation [14,15]. Previous studies have reported that activation of Notch 3 signaling promotes non-pulmonary artery smooth muscle cells proliferation by suppressing the expression of p27Kip1 [16,17]. Other Studies have demonstrated that activation of Notch3 promotes the proliferation of hepatocellular carcinoma cells through up-regulation of Hes1 expression and subsequent reduction of p27Kip1 [18]. However, it is unclear whether these molecular mechanisms are responsible for Notch3 cascade-mediated PASMCs proliferation. To address these issues, primary cultured PASMCs were transfected with adenovirus to over-express NICD3, and cell proliferation was determined, molecular mechanisms underlying this effect were further explored.

## 2 Materials and methods

### 2.1 Cell preparation and culture

Primary pulmonary arterial smooth muscle cells from pulmonary arteries were prepared from Sprague–Dawley rats (100–150 g). All animal care and experiments were performed in accordance with the Guide for the Care and Use of Laboratory Animals of Xi’an Jiaotong University Animal Experiment Center. All protocols used in this study were approved by the Laboratory Animal Care Committee of Xi’an Jiaotong University. Briefly, the isolated pulmonary arteries were rapidly removed from euthanized rats by CO_2_ overdose, washed in phosphate-buffered saline (4 °C), and then dipped into Dulbecco’s Modified Eagle Medium (DMEM, Gibco) with 10% fetal bovine serum (FBS, Sijiqing), 100 U/ml penicillin, and 100 μg/ml streptomycin (complete DMEM). A thin layer of the adventitia was carefully stripped off with fine forceps, and the endothelium was removed by gently scratching the intima surface with a surgical blade. Next, pulmonary arteries were cut into 1 mm tissue blocks and placed into a culture flask and incubated at 37 °C in an atmosphere of 95% air and 5% CO_2_ till cells reaching 80% confluence. Then cells were passaged using 0.25% trypsin. Cells between 4 and 6th passage were used in the study. To test the purity of smooth muscle cells, cells were stained with 4′,6′-diamidino-2-phenylindole (DAPI, Invitrogen) and FITC-labeled anti-smooth muscle α-actin antibody (Sigma) for nucleus and smooth muscle actin, respectively. Fluorescence microscope images indicated that cells contained more than 94% of smooth muscle cells (data not shown here). Before each experiment, cells were incubated in 1% FBS-DMEM overnight to minimize serum-induced effect on the cells, and cells viability was more than 95% determined by trypan blue staining.

### 2.2 Adenoviral transfection

NICD3 and null adenovirus were provided by Vector Biolabs (Mavelm). Transfection was performed at a multiplicity of infection of 100 plaque-forming units in 2 ml (6 mm culture dishes) DMEM for 48 h at 37 °C, 5% CO_2_ in a humidified incubator before harvesting and analysis.

### 2.3 siRNA transfection

To silence the expression of Hes1 protein, PASMCs were transfected with sequence-specific or non-targeting control siRNA (Dharmacon) using Lipofectamine™ 2000 reagent (Invitrogen) as described previously [19]. Briefly, cells were cultured until they reached 30–40% confluence; siRNA and Lipofectamine were diluted in serum-free DMEM separately and incubated for 5 min at room temperature. siRNA was mixed with Lipofectamine and incubated at room temperature for 20 min. Then, the complex of siRNA and Lipofectamine was added into cells and cells were cultured for 48 h at 37 °C, 5% CO_2_ in a humidified incubator. Effects of siRNA transfection were analyzed using Western blotting.

### 2.4 Brdu incorporation assay

To examine PASMCs proliferation, the rate of BrdU incorporation was determined using BrdU ELISA Kit (Maibio) according to the manufacturer instructions. Briefly, cells were seeded into 96-well plate at a density of 5 × 10^3^ cells/well, then BrdU labeling reagent was added to the wells and incubated for 2 h at 37 °C. Next, cells were denatured with FixDenat solution for 30 min at room temperature, and followed by incubating with anti-BrdU mAbs conjugated to peroxidase for 90 min at room temperature. After removing antibody conjugate, substrate solution was added for reaction of 10 min. The absorbance at 370 nm was determined with a microplate reader (Bio-Rad). The blank corresponded to 100 μL of culture medium with or without BrdU.

### 2.5 Western blotting

The cultured cells were washed twice with ice-cold PBS and lysed on ice with RIPA lysis buffer containing freshly added protease and phosphatase inhibitor cocktails. After 15 min of incubation, cell lysate was centrifuged for 10 min (15,000 × *g*) at 4 °C; supernatant was saved as total protein. Equivalent amounts of protein (30 μg) from each sample were separated on SDS–polyacrylamide gels, then transferred to nitrocellulose membranes (Bio-Rad). Monoclonal antibodies against Notch3 (Abcam, 1:500 dilution) and Hes1 (Cell Signaling Technology, 1:1000 dilution), p27Kip1 (Cell Signaling Technology, 1:1000 dilution) as well as polyclonal antibody against GAPDH (Sigma–Aldrich, 1:2000 dilution) were used according to the manufacturer instructions. Horseradish peroxidase-conjugated goat anti-rabbit IgG was used as secondary antibody (Sigma–Aldrich, 1:5000 dilution). Blots were developed using ECL reagent kit (Millipore). The signal intensity of the appropriate bands on the autoradiogram was calculated by using Scion Image software (Scion).

### 2.6 Statistical analysis

Data are expressed as mean ± SEM. Statistical significance was determined using one-way ANOVA followed by Tukey post hoc test. *P* < 0.05 was considered to be significant between groups.

## 3 Results

### 3.1 Over-expression of NICD3 promotes PASMCs proliferation

**Fig. 1:**
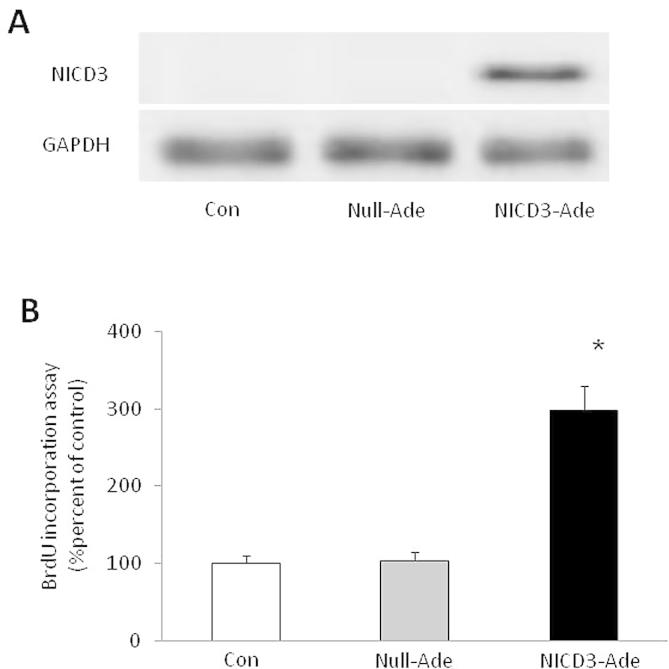
Over-expression of NICD3 induces PASMCs proliferation. (A) The representative Western blot of NICD3 over-expression by adenovirus transfection in primary cultured PASMCs (*n* = 3). (B) Over-expression of NICD3 induced PASMCs proliferation assessed by BrdU incorporation assay (*n* = 4 each group). ^*^P < 0.01 versus control cells.

To determine whether activation of Notch3 induces PASMCs proliferation, NICD3 was over-expressed in cells by adenovirus transfection. Fig. 1A shows that control PASMCs had almost no basal NICD3 protein, and null adenovirus transfection did not increase NICD3 protein level. PASMCs transfected with adenovirus carrying NICD3 showed a dramatic increase in NICD3 protein level. Fig. 1B indicates that cells with over-expressed NICD3 exhibited an elevated proliferation assessed by BrdU incorporation assay, which was 3-fold increase over control (*P* < 0.01), while transfection of cells with null adenovirus did not affect cells proliferation. These results suggest that activation of Notch3 effectively induces PASMCs proliferation.

### 3.2 NICD3 up-regulates Hes1 expression and reduces p27Kip1 expression in PASMCs

**Fig. 2:**
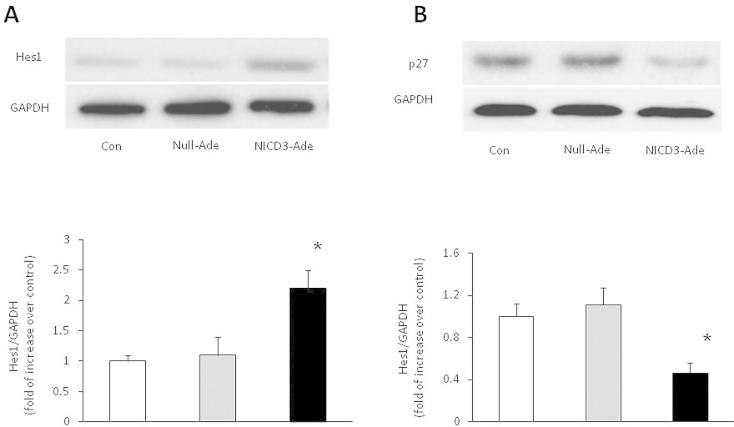
Over expression of NICD3 modulates Hes1 and p27Kip1 expression. Primary cultured PASMC were transfected with control (null) or NICD3 adenovirus for 48 h, cell lysates were used for analysis protein expression of Hes1 (A) and p27 Kip1 (B). The representative Western blot and quantification of bands are shown (*n* = 3) ^*^P < 0.01 versus control group.

To investigate the mechanisms underlying activation of Notch3 inducing PASMCs proliferation, down-stream targets of Notch3 signaling pathway and cell cycle protein p27Kip1 were determined in cells. As shown in Fig. 2A, cells transfected with NICD3 exhibited an increased Hes1 protein expression, a 2.1-fold increase over control was achieved in cells (*P* < 0.01 versus control). Null adenovirus transfection did not affect Hes1 protein level. Fig. 2B indicates that protein level of p27Kip1 was reduced in NICD3 over-expressed cells, which was 0.4-fold increase compared with control (*P* < 0.01), while null adenovirus transfection did not change p27Kip1 protein level. These results suggest that activation of Notch3 modulates Hes1 and P27Kip1 protein expression in PASMCs.

### 3.3 Hes1 mediates NICD3-induced reduction of p27Kip1 in PASMCs

**Fig. 3:**
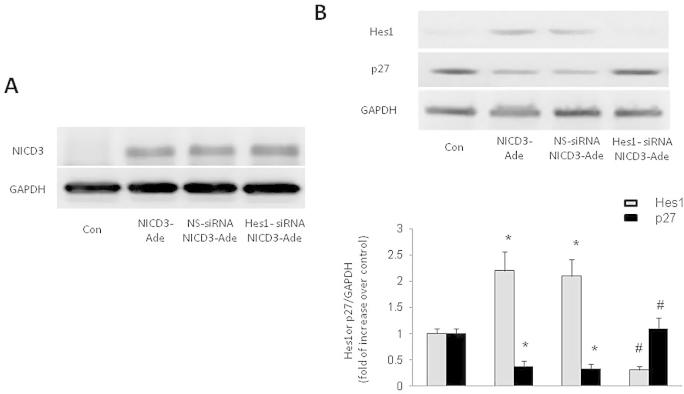
Up-regulation of Hes1 mediates NICD3-induced p27Kip1 down-regulation. (A) PASMCs were prior transfected with sequence specific or non-specific siRNA targeting on Hes1 before adenovirus carrying NICD3 infection, Western blot shows that siRNA transfection did not affect NICD3 protein expression (*n* = 3). (B) Loss of Hes1 reversed NICD3 over-expression-induced p27Kip1 protein reduction (*n* = 3). ^*^P < 0.01 versus control group; ^#^*P* < 0.01 versus NICD3 group.

To determine whether induction of Hes1 specifically mediated NICD3-induced p27Kip1 reduction, knockdown of Hes1 was applied in the study. Since the basal level of Hes1 protein is pretty low, we did not examine the silencing effect in normal PASMCs. Fig. 3A indicates that prior transfection of cells with either non-specific siRNA or Hes1 siRNA did not affect NICD3 adenovirus transfection-induced NICD3 expression. As shown in Fig. 3B, prior transfection of Hes1 siRNA inhibited NICD3-induced Hes1 expression and p27Kip1 reduction, Hes1 decreased from 2.2-fold increase over control in NICD3 transfected cells to 0.30-fold increase over control in NICD3 transfected cells with prior Hes1 silencing (*P* < 0.01), this was accompanied with the change of p27Kip1 from 0.37-fold increase to 1.1-fold increase over control (*P* < 0.01). These results suggest that up-regulation of Hes1 is responsible for over-expression of NICD3-induced p27Kip1 reduction.

### 3.4 Changes of Hes1/p27Kip1 mediated NICD3-induced PASMCs proliferation

**Fig. 4:**
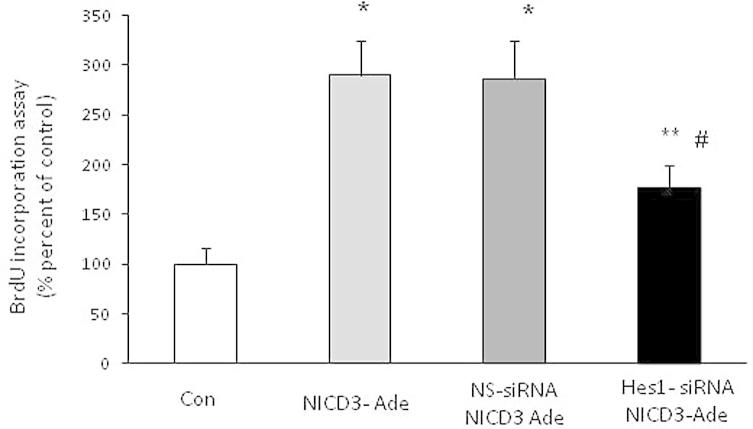
Knockdown of Hes1 blocks NICD3-induced PASMCs proliferation. PASMCs were prior silenced with Hes1 siRNA and then infected with NICD3 adenovirus, cell proliferation was determined using BrdU incorporation assay (*n* = 4). ^*^P < 0.01, ^**^P < 0.05 versus control group; ^#^*P* < 0.05 versus NICD3 group.

To examine whether the change of Hes1/p27Kip1 caused by NICD3 over-expression is associated with NICD3-induced PASMCs proliferation, BrdU incorporation was determined in cells transfected with NICD3 adenovirus with or without prior silencing Hes1. Fig. 4 shows that over-expression of NICD3 triggered a 2.90-fold increase in cell proliferation compared with control (*P* < 0.01), while knockdown of Hes1 by siRNA transfection partially suppressed cells proliferation induced by NICD3 over-expression, which decreased to 1.76-fold increase over control (*P* < 0.05 versus NICD3-transfected cells). Non specific siRNA did not affect NICD3-induced cell proliferation. These results suggest that Hes1-induced p27Kip1 reduction mediates NICD3 stimulation of PASMCs proliferation.

## 4 Discussion

In the present study, we provide direct evidence that activation of Notch3 signaling by over-expression of NICD3 stimulates primary cultured PASMCs proliferation; this effect is coupled to NICD3 up-regulation of Hes1 expression and subsequent down-regulation of p27Kip1. The present study also suggests that targeting Notch3 cascade might be a novel strategy for the management of PAH.

Notch3 receptor is predominantly expressed in arterial vascular smooth muscle cells, and modulates multiple behaviors of vascular smooth muscle cells including proliferation, apoptosis and migration [20,21]. It has been shown that Notch3-null mice is defected in the vascular development [16], and Notch3 mutations are responsible for heritable cerebral autosomal dominant arteriopathy with subcortical infarcts and leukoencephalopathy (CADASIL) syndrome with alterations in vascular smooth muscle cells (VSMCs) [22]. Recent study has indicated that Notch3 missense mutations are identified in patients with PAH and verified that these mutations are associated with PASMCs proliferation [15]. Li et al. have found that Notch3 expression is elevated in clinic patients with PAH; they further report that Notch3 knockout transgenic mice are resistant to hypoxia-induced PAH. On the other hand, inhibition of Notch3 cleavage (activation) by gamma secretase inhibitor blocks the occurrence of PAH in animal model by suppression of PASMCs proliferation [14]. Our results provided the direct evidence that over-expression of NICD3 (activation of Notch3 signaling) caused PASMCs proliferation, and further confirmed above observation.

Upon Notch3 activation, released NICD3 translocates to nucleus, where it forms complex with CBF-1/RBP-Jk to regulate target genes transcription [23] including Hes transcriptional repressors [24–27]. Hes is a group of bHLH transcription factor repressors, which negatively modulate cell cycle progression and cell proliferation by regulating transcription of cell cycle proteins [28]. Hes1 binds to the promoter of p27 Kip1and reduces p27Kip1 expression in a variety of tumor cells [12,18] and non-pulmonary vascular smooth muscle cells leading to these cells proliferation [29,30]. The present study indicated that up-regulation of Hes1 by over-expression of NICD3 also particularly mediated transcriptional suppression of p27Kip1 in PASMCs.

p27Kip1 plays a critical role in regulating cell cycle progression in mammalian cells. As a cyclin-dependent kinase (CDK) inhibitor, p27Kip1 binds to and inhibits the function of cyclin E/CDK2 complex, and further blocks cell cycle progression from G1 into S phase and suppresses cell proliferation. The present study showed that up-regulation of Hes1 was responsible for the reduction of p27Kip1 protein level in NICD3 over-expressed PASMCs; however, silencing Hes1 did not fully suppress PASMCs proliferation despite the restoration of p27Kip1 protein level, suggesting that other mechanisms independent of Hes1/p27Kip1 might be also involved in the activation of Notch3-induced PASMCs proliferation. Giving the fact that enhanced Notch3 signaling is associated with the pulmonary vascular remodeling in clinic patients and a variety of animal model of PAH, the strategy that interfering Notch3 signaling pathway might prevent or treat the development of PAH, it is worthy to test the effect and safety of this approach in clinic patients with PAH.

## Competing interests

The authors declare no competing interests.

## Author contributions

Manxiang Li designed the study; Yang Song, Yonghong Zhang and Haoxiang Jiang performed the experiments; Yanting Zhu, Lu Liu, Lan Yang and Yibin Wang analyzed and interpreted the data; Yang Song, Wei Feng and Yonghong Zhang wrote the paper.
